# Spatial variation and individual specialization of stickleback diet in relation to trophic morphology

**DOI:** 10.1007/s10452-026-10299-x

**Published:** 2026-04-24

**Authors:** Ragna Guðrún Snorradóttir, Bjarni K. Kristjánsson, Joseph Phillips, Kasha Strickland

**Affiliations:** 1 Department of Aquaculture and Fish Biology, Hólar University, Hólar Í Hjaltadal, 551 Sauðárkrókur, Iceland; 2 Northwest Iceland Nature Research Centre, Aðalgötu 2, 550 Sauðárkrókur, Iceland; 3 Department of Biology, Creighton University, Omaha, NE 68178 USA; 4 Institute of Ecology and Evolution, School of Biological Sciences, University of Edinburgh, Edinburgh, EH9 3FL UK

**Keywords:** Niche partitioning, Diet, Specialization, Threespine stickleback, Mývatn, Trophic, Morphology

## Abstract

A population’s dietary niche, including individual variation and specialization, shapes the scope and strength of its trophic linkages. Individual diet variation may emerge in response to spatial variation in the selective pressures that shape trophic morphologies, such as food availability or competition. Therefore, characterizing dietary niche variation and its link to morphological differences among individuals is a key focus in evolutionary ecology. We examined how diet composition and individual specialization varied across space and trophic morphology in threespine stickleback (*Gasterosteus aculeatus*) in Lake Mývatn, Iceland. We found that trophic morphology and diet varied spatially, which may reflect dietary niche partitioning or environmental food availability. Diet variation correlated with body size and trophic morphology: smaller fish consumed more cladocerans, while larger fish ate more chironomid larvae, mollusks, and stickleback eggs. Gut length had the next strongest relationship with diet, with longer guts linked to harder prey and shorter guts associated with stickleback eggs. Gill raker length and head shape were also associated with diet variation, suggesting functional morphological feeding differences. Most stickleback were dietary generalists, but individuals from a relatively warm habitat were more specialized than the rest of the population. More specialized individuals tended to feed more on crustaceans or mollusks, as opposed to chironomids. However, specialization was not linked to trophic traits measured in this study, implying that it is underpinned by other traits. Our findings demonstrate spatial variation in diet and specialization in Mývatn stickleback, despite the population’s generalist feeding and limited spatial genetic differentiation.

**Supplementary Information:**

The online version contains supplementary material available at 10.1007/s10452-026-10299-x.

## Introduction

The dietary niche of a population or species, defined as the types and amounts of food consumed by individuals within that population, is a central component of its broader ecological niche (Bolnick et al. 2003). It is frequently used as a proxy for the overall niche breadth of a population and its constituent individuals, reflecting the range of resources and environments they can exploit (Machovsky-Capuska et al. 2016; Sexton et al. 2017). As such, the dietary niche plays a fundamental role in shaping ecological and evolutionary processes by mediating organism–environment interactions (Kearney et al. 2010; Sultan 2015). Moreover, differentiation between species in their dietary niche has long been recognized as a key mechanism that facilitates coexistence by reducing resource competition. Such resource partitioning (also known as niche partitioning) is thought to lead to divergent selective pressures that can result in adaptations to optimize the acquisition of food sources and maximize fitness (i.e., character displacement, (Brown and Wilson 1956; Letten et al. 2017; Axelrod et al. 2018; Ellner et al. 2019)). For example, the evolution of beak morphology in Darwin’s finches on the Galápagos Islands illustrates how feeding on different seed types promoted sympatric speciation (Grant and Grant 1996). Understanding dietary niches is therefore crucial for unravelling the mechanisms underlying species interactions, community structure, and the adaptive landscapes experienced by populations.

Although niche theory was initially developed to describe species- or population-level patterns, growing evidence suggests that substantial dietary variation also exists among individuals within single populations (Van Valen 1965; Bolnick et al. 2003; Strickland et al. 2021). Some researchers have extended the principles of niche partitioning to the individual level, proposing that individuals within a single population may occupy distinct dietary niches (Costa-Pereira et al. 2018, 2019; Ingram et al. 2018). These differences expose individuals to unique selective pressures, potentially resulting in character displacement among subsets of individuals and contributing to adaptive divergence within populations (Schluter and McPhail 1992). Individual variation in dietary niche within populations may be an important mechanism that underlies how adaptive divergence and local adaptation occurs within populations, and therefore is of central interest to evolutionary ecologists.

A central aspect of individual-level dietary variation is the degree of dietary specialization. Much like the generalist-specialist axis used to describe species dietary niches, individuals within the same population can vary widely in the breadth of their diet (Bolnick et al. 2003). Some individuals act as generalists, consuming a broad range of food items, while others are specialists, focusing on a narrower subset of resources (Svanbäck and Bolnick 2006). This phenomenon, known as “individual specialization”, has been found across multiple taxa (Bolnick et al. 2003; De León et al. 2012; Cardona et al. 2017). The level of dietary specialization can strongly affect ecological processes, such as community and food web structures, as well as evolutionary processes, such as the extent of gene flow and strength or modes of natural selection experienced by groups of individuals (Bolnick et al. 2011). As such, uncovering the causes and consequences of individual dietary variation is vital to our understanding of both population-level evolution and ecosystem functioning.

Dietary differences among individuals are often linked to variation in trophic morphology, forming a critical part of an organism’s multidimensional ecological niche (Machovsky-Capuska et al. 2016; Ventura et al. 2017). These individual differences can arise from a combination of ecological and intrinsic factors. For instance, resource availability, competition, predation, and parasitism can all influence dietary strategies (Araújo et al. 2011; Britton and Andreou 2016), but it is also impacted by intrinsic factors like ontogenetic niche shifts, sexual dimorphism, and phenotypic variation (Svanbäck and Bolnick 2006). For example, diet variation within populations may reflect spatial differences in intraspecific competition and resource availability, but individuals also choose their diet based on their trophic morphology or other traits (Svanbäck and Persson 2004). These differences in feeding and trophic morphologies affect foraging strategies, ultimately determining the types of food an individual can exploit (Ferry-Graham et al. 2002). As such, we expect that within-population variability in diet will be both variable across space and be linked to individuals’ trophic morphology.

In this study, we characterized individual variation in diet and trophic morphology across ecologically divergent sites for a population of threespine stickleback (*Gasterosteus aculeatus*) in Lake Mývatn in Northeast Iceland. The lake is spatially heterogenous, driven by its complex geomorphology and groundwater springs that vary in temperature and chemistry (Einarsson et al. 2004). Mývatn’s biological populations are temporally variable, demonstrated by temporal fluctuations in chironomid midges, stickleback, arctic charr (*Salvelinus alpinus*) and piscivorous birds (Einarsson et al. 2004; Ives et al. 2008; Millet et al. 2013). Stickleback are found across the entire lake and are highly panmictic (Strickland et al. 2023), and thus constitute a demographically integrated metapopulation subject to a wide range of ecological conditions (Phillips et al. 2023). Despite extensive genetic admixture, prior work has demonstrated both spatial variation in trophic morphologies of Mývatn stickleback, and temporal change in these traits that was linked to genomic signatures of directional natural selection (Millet et al. 2013; Strickland et al. 2023, 2024). Moreover, there is some evidence to suggest that stickleback diet may vary between specific areas of the lake (Koopmans 2010), which could be associated with observed spatial variation in trophic morphology. Together, this body of research suggests that the dietary niche is likely key to both evolutionary and ecological processes in Mývatn stickleback, making this system ideal for studying spatial variation of morphology and diet, as well as their relationship, and how these relate to individual specialization within the ecosystem.

We sampled stickleback from across Mývatn to (1) characterize spatial variation in morphology, diet and individual specialization in diet, and (2) determine if individual variation and/or specialization in diet is associated with trophic morphology. By investigating the connection between diet, morphology, and specialization in threespine stickleback, this study seeks to enhance our understanding of the ecological and evolutionary processes shaping trophic adaptations. We hypothesize that individual variation in diet reflects both ecological differences across sites and the underlying selective pressures, and that these dietary patterns are associated with specific trophic morphological traits. This research will shed light on the role of ecological variation in driving morphological diversity and specialization in natural populations.

## Materials and methods

### Study system and stickleback sampling

Mývatn (65° 36′ N, 17° 00′ W; 37 km^2^), Northeast Iceland, is geologically young (formed about 2300 years ago) and highly environmentally heterogeneous (Einarsson et al. 2004; Millet et al. 2013). The lake is divided into two basins: the north (ísl. Ytri-flói) and the south (ísl. Syðri-flói) (Jónasson 1979). The south basin is substantially larger (28.5 km^2^) than the north (8.5 km^2^), and is also shallower (max depth ~ 4 m vs. 7 m) due to diatomite mining in the north basin between 1967 and 2004 ((Einarsson et al. 2004); unpublished data). The lake is fed by springs along the eastern shoreline, varying in temperature from as low as 5 °C in the south to 30 °C in the north (Jónasson 1979). The basins also differ in various biotic characteristics, such as vegetation type, zooplankton, phytoplankton, and in densities of chironomid midge larvae and other benthic organisms, stickleback, and birds (Jónasson 1979; Einarsson et al. 2004; Millet et al. 2013). Threespine stickleback are substantially more abundant in the north basin despite its smaller area, with the north basins sub-population subsidizing the south basin and driving lake-wide population dynamics (Phillips et al. 2023).

**Fig. 1:**
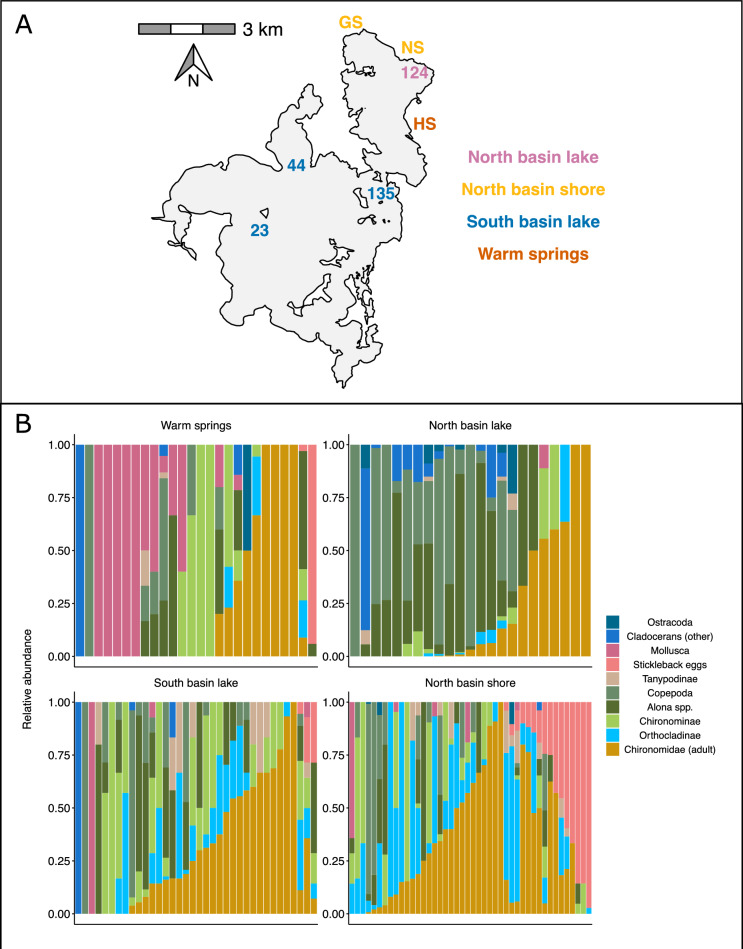
**A** Map of sampling locations around Mývatn, with colors denoting groupings for analysis, and **B** a stacked barplot showing the relative abundances of different taxa in the diet of individual stickleback. Each bar is an individual and individuals with empty stomachs not shown, and each panel depicts individuals caught at the different sampling locations

The stickleback population in Mývatn has been extensively researched as part of a long-term monitoring program spanning almost 40 years (Gíslason et al. 1998; Phillips et al. 2023). For this study, we sampled stickleback on 17th and 18th of June 2021 from three shoreline sites in the north basin (GS, NS, and HS), one offshore “lake” site in the north basin (124), and three lake sites in the south basin (23, 44, and 135) (Fig. 1A). We acknowledge that sampling from a single month in a single year provides a “snapshot” that does not fully encompass the dynamic nature of the food available for sticklebacks to consume. However, limiting the temporal scope of our sampling allowed us to maximize our sample sizes for a single time point, as appropriate to our focus on spatial variation and diet-morphology associations. The sites were selected to represent the ecological variation and different habitats that occur within the lake (see (Millet et al. 2013; Strickland et al. 2023)). To ensure suitable sample sizes, we pooled ecologically similar sites for all downstream analyses: north basin lake (124; relatively deep and soft substrate), south basin lake (23, 44, and 135; relatively shallow and soft substrate), north basin shore (GS and NS; shallow and mix of rocky and soft substrate), and warm springs (HS; a warm-spring fed embayment with soft substrate). Fish were caught in unbaited minnow traps, laid for 2–12 h during the day, with five traps at each site. The trap durations were intended to be as short as possible to minimize digestion in the stomachs of the fish, while being long enough to ensure enough individuals were sampled. We euthanized fish immediately upon capture by chilling and then freezing. Specimens were then placed in 96% ethanol as soon as possible (normally within 90 min). We replaced the ethanol after 72 h and left the specimens to fix for between 50 and 55 days prior to further analysis.

### Diet and trophic morphology

For diet and morphology identification, 50 individuals were randomly selected from each site, apart from the two lake sites in the south basin where there were only nine fish caught at each site (and so all fish were included). We selected adult fish by choosing those equal to or larger than 35 mm, which are considered to be late juveniles or adults, having developed fully adult bony structure (Bell 1982). This resulted in a total of 268 individuals. For each fish, we then identified their diet based on stomach contents and measured a suite of morphological traits which are thought to predict stickleback diet (Day and McPhail 1996; Walker 1997; Bolnick and Paull 2009; Matthews et al. 2010). These traits were total length, gut length, length of gill rakers, gap width between gill rakers and head shape (detailed below). Gut length has previously been linked to diet in fish, and may describe the general feeding preference of an individual (Karachle and Stergiou 2010). Generally, fish that prey on items that are resistant to digestion, like plant material and crustacean exoskeletons, have a relatively longer gut and fish with a relatively shorter gut may be associated with a more carnivorous diet of softer bodied animals. The gill raker structure is a key trophic phenotype that dictates the type of foods that a stickleback may eat. For instance, the length of the gill rakers and the width between gill rakers have been shown to relate to limnetic and benthic diets (Matthews et al. 2010). Gill raker structure and head shape have also been shown to be linked to diet (Lavin and McPhail 1985; Day and McPhail 1996).

For each fish, we measured total body length (to the nearest mm) and mass (± 0.001 g), and we then photographed the left side of the fish (Canon EOS 250D, with a 50 mm macro lens) for morphometric analyses (described below). We then dissected the fish to remove the entire gut (including the stomach) and determined the sex through visual examination of gonads. Gut length was measured (to the nearest mm) from the sphincter at the end of the esophagus to the end of the digestive tract using a Vernier caliper. Next, the stomach contents were emptied into a petri dish with 96% ethanol. Using a dissecting microscope (Leica M165 C), we identified and counted the stomach contents to the finest feasible taxonomic resolution. Chironomidae larvae were identified to either subfamily (Orhocladiinae, Tanypodinae) or Tribe (Chironomini and Tanytarsini), based on head capsule morphology. Adult and pupae of all chironomids were grouped together. Most cladocerans were identified to species based on the carapace, including *Acroperus harpae*, *Alonella nana*, *Daphnia longispina*, and *Macrothrix hirsuticornix*. The cladoceran genus *Alona* contains three species at Mývatn (*A*. *affinis, A. quadrangularis*, and A*. rectangula*), however, we did not attempt to distinguish between *A*. *affinis* and *A. quadrangularis*. There were some Cladocera remains that were extensively degraded and were therefore categorized as “unknown” Cladocera. Other diet items included copepods (separately identified as nauplii and adults), ostracods, gastropods, bivalves, and stickleback eggs. We also noted the presence of plant material, although this was not included in the analysis. As some taxa were found in a small proportion of individuals, we aggregated these to ensure reasonable representation across the data set for our statistical analysis, resulting in the following taxonomic groups: Adult chironomid, Orthocladiinae, Chironominae, Tanypodinae, *Alona spp.*, Cladoceran (except *Alona*), copepod, ostracod, mollusk, and stickleback eggs (see Table S1).

After the initial dissection, the fish were stained to highlight bony characteristics. This was done by first bleaching the fish with a solution made up of 60% potassium hydroxide (1% KOH) and 40% hydrogen peroxide (3% H_2_O_2_) solution. When the fish were white and their eyes were brown, they were rinsed with 1% KOH and the color solution (alizarin red in 1% KOH) was added. After around 1–2 h of being in the solution, they were removed and a 1% KOH solution was added to rinse the color solution from the fish. Stained fish were then rinsed overnight in cold water. After staining, the first gill raker from the left side of the fish was removed under a dissecting microscope (Leica M165 C). The gill raker was placed between two glass plates in a drop of ethanol that fully immersed the gill arch and photographed, with a mm scale, under a dissecting microscope (Leica M165 C with Leica MC170 HD camera), then labelled and stored in 96% ethanol. The length of the second and third long gill raker and the gap width between them were measured from photographs in ImageJ (Schneider et al. 2012). We measured the second and third gill raker, rather than the first, because the first fill raker was sometimes broken during dissection. To reduce measurement error, three measurements were taken of each structure and the average used. In ImageJ, measurements can be taken with straight and segmented lines. Straight lines were used to measure the width between gill rakers, while segmented lines to measure the gill raker length to account for curvature.

We used geometric morphometrics to characterize the head morphology of each fish. Digital photographs taken prior to dissection (as described above) where landmarked using the software tpsDig2 (version 2.32) (Rohlf 2015). We digitally placed 33 landmarks on the head of each specimen with 10 “fixed” landmarks on specific morphological features and 23 “sliding” landmarks along the contours of the head. Landmarks from previous studies on geometric morphometrics on stickleback were used for reference for the selection of landmarks (Kristjánsson 2005; Taugbøl et al. 2014; Jakubavičiūtė et al. 2018), furthermore additional 20 sliding landmarks placed between the anterior-most point of the orbital and between the tip of the snout and the anterior end of the supraoccipital bone to estimate how the curve of the head can vary, which has been shown important in relation to feeding in previous studies (Parsons et al. 2010).

Head morphology was analyzed using the package *geomorph* (Adams and Otárola-Castillo 2013) in R version 4.5.1 (R Core Team 2021). Landmarks were subjected to a generalized Procrustes transformation to remove isometric effects of size on shape, as well as effects of rotation of the fish and position on the photograph. We then conducted a Principal Components Analysis (PCA) to describe individual variation in the shape described by these landmarks, using the *gpagen* function in *geomorph*. This function scales variables by their standard deviations and centers on their means prior to analysis. We use the first three PCA axes for further analysis (see below). To visualize the morphological variation in relation to these axes we used the function *plotRefToTarget* to visualize the morphology of fish at the extreme ends of the PCA axes in relation to the mean morphology of the fish.

### Statistical analysis

While we measured diet and morphology from 268 fish, some variables could not be measured for every fish. Specifically, some guts, stomach contents, and gill rakers were damaged or incompletely preserved following dissection, and only individuals that were at most slightly bent following fixation could be landmarked. Therefore, the final dataset included 206 individuals for which we had data on both diet and morphology that were included in downstream analyses.

#### Spatial variation in trophic morphology

To characterize spatial variation in trophic morphology we fit a multivariate mixed effects model in a Bayesian framework using the R package ‘brms’ (Bürkner 2017), which is a wrapper for the ‘rstan’ package (Stan Development 2018). This model fit the seven morphological traits as a multivariate response variable (body length, gut length, gill raker length, gill raker gap width, PC1, PC2, PC3) with sex treated as a fixed effect for all traits. Body length was included as a fixed covariate for the six other traits as they are expected to allometrically scale with body size, such that spatial variation and covariation among traits factored out the effect of body size. Site was fit as a random effect to estimate spatial variation in each trait after controlling for sex and allometry. Full variance–covariance matrices were estimated for the site random effect, as well as for the residual term. All traits were z-transformed (subtracted mean and divided by standard deviation), and the model was fit using Gaussian error distributions for all traits. We fit the model with four chains run for 4000 iterations, including a 2000-iteration adaptation period and using default priors. Convergence was assessed using the potential scale reduction factor and effective sample size, following the default thresholds specified in ‘brms’.

#### Spatial differences in diet and relationship between diet and morphology

We used a generalized linear mixed model (GLMM) to quantify taxon-specific associations between dietary abundance and stickleback morphology, while also accounting for dietary variation across sites, using ‘brms’ as above. The model was fit to the data in a long format, such that each row of the data corresponded to the observed abundance of a particular taxon in the stomach of an individual fish, repeated for all taxon-by-individual combinations (following (Jackson et al. 2012)). We used the raw counts as the response variable, modelled with a zero-inflated Poisson error distribution and a log link-function. The model included fixed effects for sex (female vs. male) and each of the morphological predictors: body length, gill raker length (averaged between the 2nd and 3rd gill rakers), gill raker gap width, gut length, and the first three axes from the principal components analysis of head landmarks. Scatter plots of the raw data against each trait are shown in the Figs. S2–S8. Gill raker length, gap width, and gut length were standardized by overall body length by using the residuals from separate linear regressions for each trait (this was not necessary for the axes from the landmark analysis, as these already accounted for body size effects). All morphological predictors were z-scored so that they could be interpreted as effect sizes on a log scale. We acknowledge that the use of principle components axes of head landmarks as predictors in downstream analyses omits potentially relevant shape variation and can be anticonservative, which can potentially be addressed by semiparametric or nonparametric approaches. However, the GLMM allowed us to explicitly model the structure of the dietary abundance data (i.e., via the zero-inflated Poisson error distribution and log link-function). Moreover, the GLMM enabled the use of random effects to characterize variation among taxonomic response to the predictors, which has the advantage of “shrinking” variation towards the mean response and reducing noisiness of the corresponding estimates.

The fixed effects of the GLMM characterized overall associations between the morphological traits and dietary abundance regardless of taxon. The random effects included “random slopes” for each morphological predictor grouped by taxon, characterizing the variation among taxa in their associations with these predictors as well as providing estimates of taxon-specific slopes. The random effects also included “random intercepts” grouped by the taxon, site, taxon × site interaction, taxon × sex interaction, and the identity of the individual fish. Because the intercepts and slopes were estimated on the linear predictor scale, the random intercepts grouped by taxon standardized the scale of variation across taxa such that the random slopes were comparable among them. Moreover, the random intercepts accounted for both overall and taxon-specific variation in abundance across sites, which corrected for the spatial variation in the availability of different food sources. The random intercepts grouped by taxon × sex interaction accounted for taxon-specific between sexes, while the random intercepts grouped by fish identity accounted for variation among individuals in the total number of prey items consumed. Finally, the zero-inflated component of the response distribution was itself modelled with random intercepts grouped by taxon (estimated with a logit-link function) to allow for variation in the probability of different diet items being completely absent from the diet of individuals across all sites. Numeric predictors were standardized and scaled as described above. Note that the random slopes were specified so that the model did not explicitly estimate correlations across the different traits grouped by taxon, to avoid identifiability issues that occurred when using a more conventional “random slopes” parameterization. The model structure in ‘brms’ syntax was specified as follows:$$\begin{gathered} Dietary{\text{ }}abundance{\text{ }}\sim {\text{ }}Length{\text{ }} + {\text{ }}Gut{\text{ }}length{\text{ }} + {\text{ }}Gap{\text{ }}width{\text{ }} + {\text{ }}Raker{\text{ }}length{\text{ }} + \hfill \\ ~~~~~~~~~~~~~~~~~~~~~~~~~~~~~~~~~~~PC1{\text{ }} + {\text{ }}PC2{\text{ }} + {\text{ }}PC3{\text{ }} + {\text{ }}Sex \hfill \\ ~~~~~~~~~~~~~~~~~~~~~~~~~~~~~~~~~~~\left( {1{\text{ }}|{\text{ }}Taxon{\text{ }} + {\text{ }}Site{\text{ }} + {\text{ }}Taxon{\text{ }}:{\text{ }}Site{\text{ }} + {\text{ }}Taxon{\text{ }}:{\text{ }}Sex{\text{ }} + {\text{ }}FishID} \right){\text{ }} + \hfill \\ ~~~~~~~~~~~~~~~~~~~~~~~~~~~~~~~~~~~\left( {0{\text{ }} + {\text{ }}Length{\text{ }}|{\text{ }}taxon} \right){\text{ }} + \hfill \\ ~~~~~~~~~~~~~~~~~~~~~~~~~~~~~~~~~~~\left( {0{\text{ }} + {\text{ }}Gut{\text{ }}length{\text{ }}|{\text{ }}taxon} \right){\text{ }} + \hfill \\ ~~~~~~~~~~~~~~~~~~~~~~~~~~~~~~~~~~~\left( {0{\text{ }} + {\text{ }}Gap{\text{ }}width{\text{ }}|{\text{ }}taxon} \right){\text{ }} + \hfill \\ ~~~~~~~~~~~~~~~~~~~~~~~~~~~~~~~~~~~\left( {0{\text{ }} + {\text{ }}Raker{\text{ }}length{\text{ }}|{\text{ }}taxon} \right){\text{ }} + \hfill \\ ~~~~~~~~~~~~~~~~~~~~~~~~~~~~~~~~~~~\left( {0{\text{ }} + {\text{ }}PC1{\text{ }}|{\text{ }}taxon} \right){\text{ }} + \hfill \\ ~~~~~~~~~~~~~~~~~~~~~~~~~~~~~~~~~~~\left( {0{\text{ }} + {\text{ }}PC2{\text{ }}|{\text{ }}taxon} \right){\text{ }} + \hfill \\ ~~~~~~~~~~~~~~~~~~~~~~~~~~~~~~~~~~~\left( {0{\text{ }} + {\text{ }}PC3{\text{ }}|{\text{ }}taxon} \right) \hfill \\ Zero{\text{ }}inflation{\text{ }}rate{\text{ }}\sim {\text{ }}\left( {1{\text{ }}|{\text{ }}taxon} \right) \hfill \\ family{\text{ }} = {\text{ }}zero\_inflated\_poisson\left( {\mathrm{``}log\mathrm{''}} \right) \hfill \\ \end{gathered}$$

The model was fit with four chains run for 4000 iterations, including a 2000-iteraction adaptation period and a thinning interval of one iteration, the “adapt delta” parameter set to 0.99 to avoid divergences, and using default priors. As above, convergence was assessed using the potential scale reduction factor and effective sample size, following the default thresholds specified in ‘brms’.

To describe the covariance structure in the presence of taxa in the diet of individual fish and test how a multivariate diet varied through space we used a constrained ordination via the “vegan” R package (Dixon 2003). To do this, we conducted a redundancy analysis (RDA) using the ‘rda’ function which considered abundances of all taxa as the response variable with site and body length fitted as explanatory variables. Abundance of taxa within each individual’s diet was scaled to unit variance within the RDA. The dataset used in RDA analyses excluded individuals with empty stomachs and were therefore run on a dataset of 128 individuals.

#### Individual specialization in dietary niche across and within sites and in relation to morphology

We used the stomach content data to quantify the extent of individual specialization and to determine whether there were differences in the level of individual specialization across sites within the lake, using the *RInSp* package in R (Zaccarelli et al. 2013). These analyses were conducted using a dataset that excluded individuals with empty stomachs and were therefore run on a dataset of 128 individuals. First, we quantified the total dietary niche width of the stickleback population (total niche width or “TNW”), measured as the variance in dietary abundance of each prey item across all individuals in the dataset (Bolnick et al. 2002). Then, we partitioned the total niche width into its within- and between-individual components (WIC and BIC respectively). The ratio of WIC to TNW quantifies the extent to which the dietary variation encompassed by whole population arises from either generalist individuals (with similar diets) or specialist individuals (with different diets) (Bolnick et al. 2002). When this ratio is close to one, all individuals feed on all diet items in similar proportions. Conversely, when this value is close to zero, individuals feed on diet items in dissimilar proportions, with some diet items present within the population as a whole excluded by some individuals. To explore whether the extent of specialization differed across space, we performed the above analysis for (1) all individuals across the lake, and (2) individuals at different sites in the lake. We then used the proportional similarity index (*PS*_*i*_) calculated for each individual (Bolnick et al. 2002) to determine whether individual specialization varied across space and in association with morphology. To do so, we fit linear models where individuals *PS*_*i*_ was modelled as a function of all morphological traits and the site at which an individual was captured. This model was fit using the *brms* in R (as above), with four chains run for 4000 iterations, including a 2000-iteration adaptation period and a thinning interval of one iteration, and using default priors. To visualize the association between individual specialization and particular diet items, we re-ran the RDA described in the previous section, but this time including *PS*_*i*_ as an additional variable.

## Results

### Geometric morphometrics

**Fig. 2:**
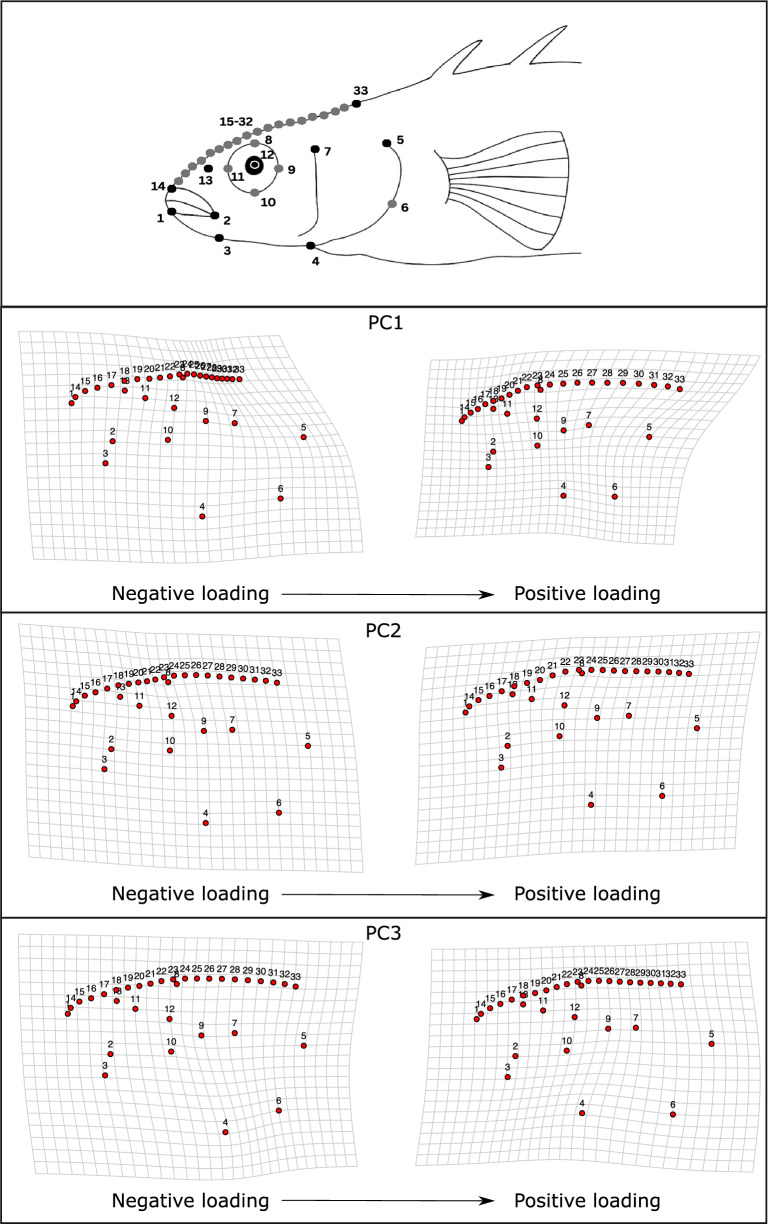
Deformation grids showing the results of geometric morphometric analyses describing the head shape of threespine stickleback from Lake Mývatn derived from landmarks illustrated in the top panel. The figure shows how head shape varies across each PC axis, and the deformation grids show the overall head shape at the extreme of first three axes from a Principal Component Analysis (indicated as negative and positive loading)

The first three PC axes characterized 69.6% of the observed head landmark variation. PC1 (35.9% of variation) broadly characterized elongation of the anterior and posterior region of the head. Specifically, more positive loadings of PC1 indicated an elongated snout and dorsal head, but smaller operculum, and relatively longer dorsal portion of the head behind the eye (Fig. 2). For PC2 (25.75% of variation), positive loadings corresponded to a longer and shallower head, while negative loadings correspond to a broader posterior (Fig. 2). Finally, PC3 (7.95% of variation) characterized the size and shape of the operculum, with positive loadings indicating a larger and more arched operculum (in terms of its curvature) (Fig. 2).

### Spatial variation in morphology

**Fig. 3:**
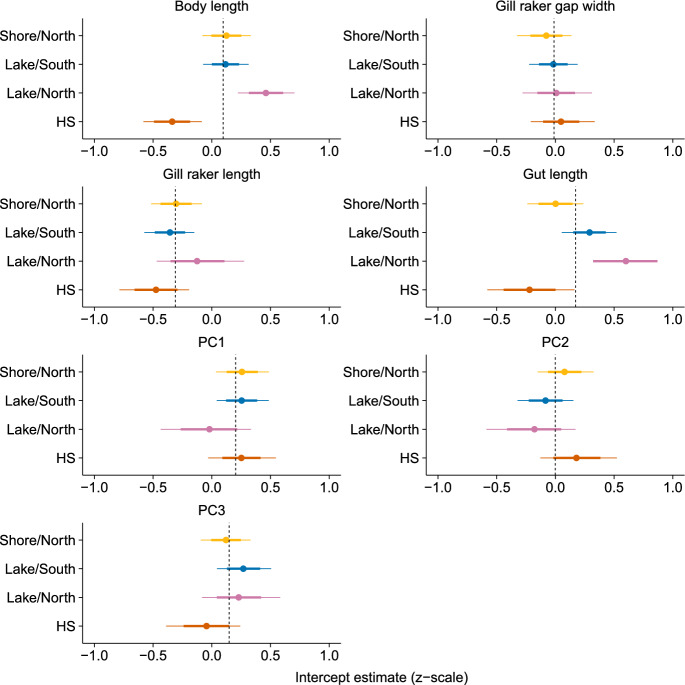
Phenotypic variation through space estimated with a multivariate GLMM. Estimates correspond to “random intercepts” grouped by site. Points indicate posterior medians of the estimated relative trait value with the error bars depicting 68% and 95% posterior uncertainty intervals (based on quantiles; 68% intervals match the coverage of standard errors). The vertical dashed lines correspond to the estimated intercept for each trait

The extent of spatial variation in stickleback traits was highly dependent on the trait (Table 1, Fig. 3). Body length was the most variable across space whereas gill raker gap width was the least (Fig. 3). As shown previously (Millet et al. 2013; Strickland et al. 2023), stickleback were shortest at the warm spring site and longest at the north basin lake sites, with intermediate body lengths at south basin lake sites and north basin shore sites. This pattern was also true for relative gut length. There was some evidence for spatial variation in head morphology (random effects standard deviations in Table 1), although it was quite subtle (Fig. 3). Individuals from the north basin lake sites had relatively more negative loadings for PC1, indicating that they had a larger operculum, shorter heads behind the eye and a rounder, larger snout. Individuals from the warm springs has relatively positive loadings for PC2, indicating longer back of the head is deeper with negative loadings, and the back of the head shorter. Conversely, individuals from the warm springs had relatively more positive loadings for PC3 indicating a relatively smaller and more open operculum (Figs. 2 and 3).

**Table 1:** Summary table of multivariate LMM used to identify spatial variation in trophic morphology. Parameter estimates are posterior medians of fixed effect coefficient estimates and standard deviations for random effects, with 95% posterior uncertainty intervals (based on quantiles) in parentheses. All traits were z-transformed so parameter estimates represent effect sizes on that scale

Trait	Fixed effect estimates			Random effects (SD)	
	Intercept	Sex_M_	Body length	Site	Residual
Body length	0.10 (− 0.43; 0.66)	− 0.26 (− 0.50; − 0.02)		0.62 (0.21; 1.4)	0.96 (0.88; 1.04)
Gut length	0.16 (− 0.44; 0.76)	− 0.45 (− 0.76; − 0.12)	0.11 (− 0.62; 0.93)	0.68 (0.14; 1.57)	1.03 (0.87; 1.39)
Gill raker length	− 0.32 (− 0.67; 0.04)	0.94 (0.64; 1.26)	0.33 (− 0.33; 0.94)	0.35 (0.02; 1.01)	1.04 (0.88; 1.29)
Gill raker gap width	− 0.01 (− 0.31; 0.28)	0.05 (− 0.26; 0.36)	− 0.09 (− 0.64; 0.43)	0.26 (0.02; 0.8)	1.07 (0.96; 1.24)
PC1	0.19 (− 0.18; 0.56)	− 0.60 (− 0.91; − 0.30)	− 0.36 (− 0.44; 0.30)	0.38 (0.03; 1.04)	1.05 (0.92; 1.26)
PC2	0.01 (− 0.40; 0.39)	− 0.02 (− 0.35; 0.30)	− 0.37 (− 1.04; 0.31)	0.4 (0.04; 1.12)	1.09 (0.94; 1.35)
PC3	0.14 (− 0.24; 0.51)	− 0.44 (− 0.73; − 0.14)	0.44 (− 0.16; 1.02)	0.37 (0.03; 1.06)	1.01 (0.88; 1.23)

### Spatial differences in diet and relationship between diet and morphology

Chironomids were the most prevalent diet item overall, with adults found in 44% of stomachs, Orthocladiinae larva in 29%, and Chironominii larvae in 27% (Fig. 1B). Other common taxa included cladocerans of the genus *Alona* and adult copepods, with mollusks generally being rarer than the chironomids and crustaceans (Fig. 1B). Note that while the chironomid group Tanytarsini was very rare, this is due at least in part to their near absence from the lake at the time of sampling (unpublished data), despite being the most numerous secondary consumer in other years (Lindegaard and Jónasson 1979). Taxa varied widely in their zero-inflated probability as estimated from the GLMM, ranging from around 11% for adult chironomids to 84% for ostracods (Fig. S9). According to the RDA, taxa showed clear patterns of covariation across individuals (Fig. S1), with positive associations among chironomid taxa and stickleback eggs, positive associations among crustaceans of all types, and mollusks being differentiated relative to the other taxonomic groups.

**Fig. 4:**
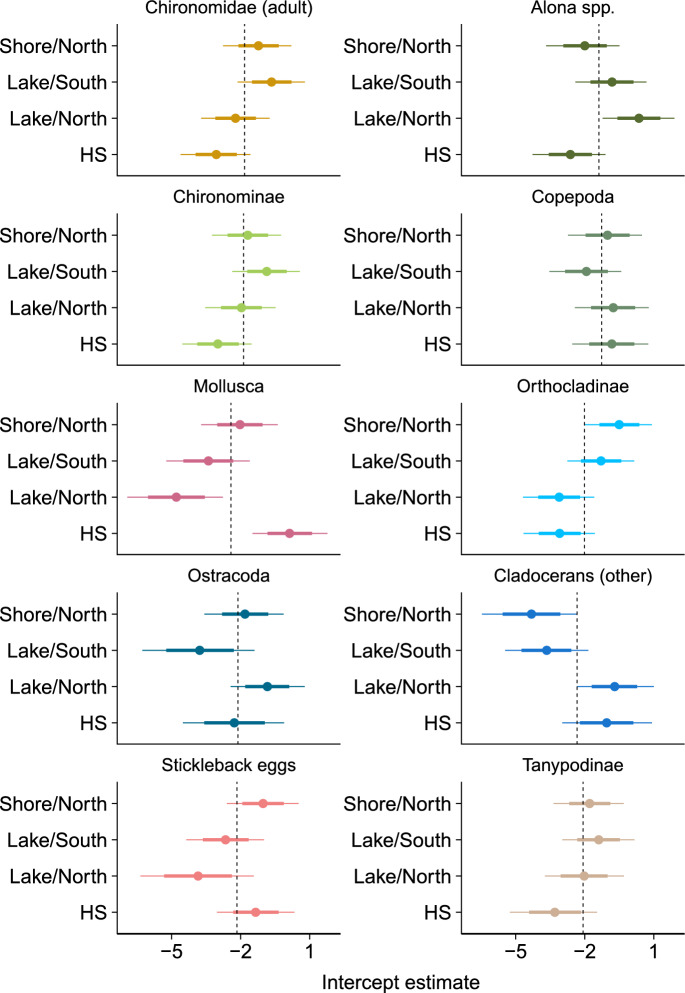
Taxon-specific variation in dietary abundance across locations, as estimated from a GLMM. Estimates correspond to “random intercepts” grouped by taxon and site. Points indicate posterior medians, with the error bars depicting 68% and 95% posterior uncertainty intervals (based on quantiles; 68% intervals match the coverage of standard errors). The vertical dashed lines correspond to the estimated intercept for each taxon

**Fig. 5:**
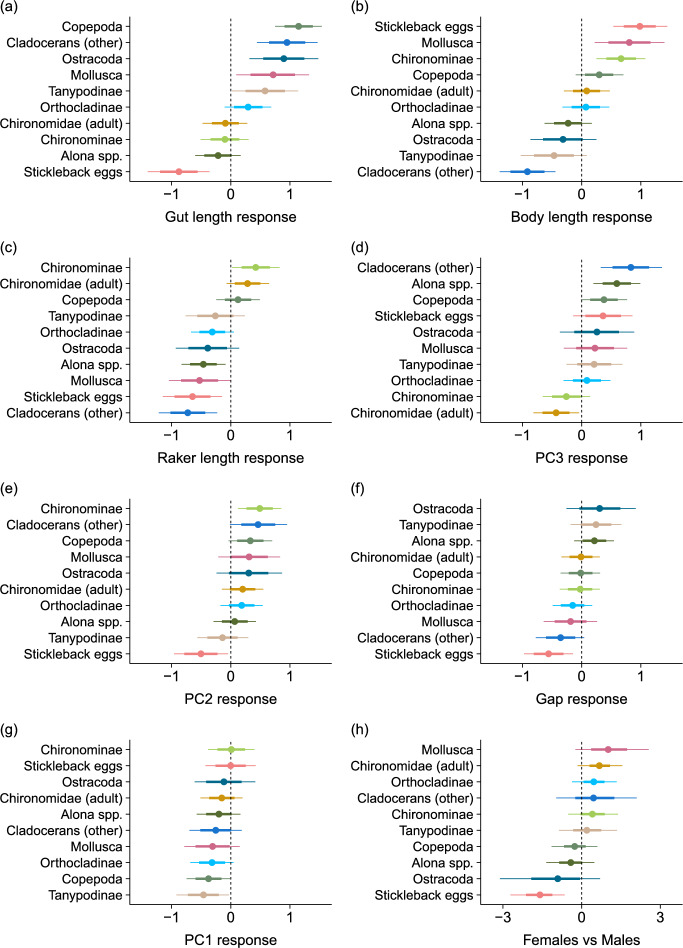
Taxon-specific responses of dietary abundance to stickleback morphology (**a**–**g**) and sex (**h**), as estimated from a GLMM. Responses in (**a**–**g**) correspond to the random effect component of the “random slope” for each morphological predictor grouped by taxon. In these panels, the x-axis scales are the same to facilitate comparison among them as effects sizes. Responses to sex (**h**) are depicted as the difference in the random intercept between females and males for each taxon, and the x-axis scale is not directly comparable to the other panels. Points indicate posterior medians, with the error bars depicting 68% and 95% posterior uncertainty intervals (based on quantiles; 68% intervals match coverage of standard errors). In each panel, taxa are ordered according to point estimate values to facilitate visualization

The total number of diet items consumed (regardless of taxon) varied widely among individuals, but there was limited evidence for variation in the number of diet items among sites and in conjunction with specific morphological characteristics (Table 2). In contrast, taxonomic diet composition varied substantially among sites and in association with morphology. Mollusks were the most obviously differentiated across sites, with by far the greatest representation in the warm springs (Table 2, Figs. 1B and 4). Dietary abundances of cladocerans (including *Alona spp.*), ostracods and stickleback eggs were modestly variable among sites, while copepods and the chironomids were relatively evenly distributed. Among the morphological characteristics, diet composition varied most in association with gut length, with crustaceans (except *Alona spp.*) and mollusks broadly being associated with longer guts, chironomids with intermediate gut lengths, and stickleback eggs with the shortest guts (Table 2, Fig. 5). Overall body length displayed a similar degree of variation among taxa, with larger individuals feeding relatively more heavily on stickleback eggs, mollusks, and Chironominae larvae (comprising primarily the large-bodied *Chironomus*, Table S1), and smaller individuals feeding more heavily on cladocerans (Fig. 5). Traits directly pertaining to trophic morphology had more modest associations with diet composition. Shorter gill rakers were strongly associated with greater representation of cladocerans and stickleback eggs, while longer gill rakers were modestly associated with greater representation of Chironominae larvae. Positive values of PC3, indicating larger and more arched opercula, were most strongly associated with cladocerans and copepods, while less positive (and negative) values were associated with chironomid larvae and adults. Moreover, negative values of PC2 (smaller shallower head) and smaller gill raker gap width were most strongly associated with stickleback eggs. In contrast, PC1 (negative values related to shorter snout, dorsal head and larger operculum) was not strongly associated with variation in diet composition. Finally, males and females had generally similar diets, aside from the fact that stickleback eggs were much more abundant in the diets of males than females, while mollusks were somewhat more abundant in females than in males.

**Table 2:** Summary table of GLMM used to quantify diet variation across space and in response to trophic morphology. Parameter estimates correspond to posterior medians, with 95% posterior uncertainty intervals (based on quantiles) in parenthesis. Continuous predictor variables were z-scored before model fitting, such that estimates represent effect sizes on that scale

	Fixed effect	Random effect group	Random effect SD
Intercept	–1.96 (–3.37; –0.70)	Individual	2.51 (2.12; 2.97)
		Site	0.61 (0.02; 2.27)
		Taxon	0.72 (0.03; 1.80)
		Taxon × Sex	0.76 (0.39; 1.36)
		Taxon × Site	1.52 (1.07; 2.11)
Sex_M_	1.17 (–0.07; 2.46)		
Body Length	0.10 (–0.54; 0.77)	Taxon (random slopes)	0.73 (0.42; 1.27)
Gut Length	0.33 (–0.35; 1.03)	|	0.76 (0.44; 1.34)
Gill Raker Gap Width	–0.05 (–0.52; 0.40)	|	0.35 (0.16; 0.66)
Gill Raker Length	–0.25 (–0.78; 0.27)	|	0.50 (0.28; 0.89)
PC1	–0.22 (–0.68; 0.23)	|	0.23 (0.08; 0.50)
PC2	0.17 (–0.32; 0.66)	|	0.39 (0.20; 0.72)
PC3	0.22 (–0.32; 0.77)	|	0.47 (0.27; 0.84)
Zero–inflation	0.19 (–0.64; 0.96)	|	1.22 (0.70; 2.13)

### Individual specialization in dietary niche across and within sites and in relation to morphology

**Fig. 6:**
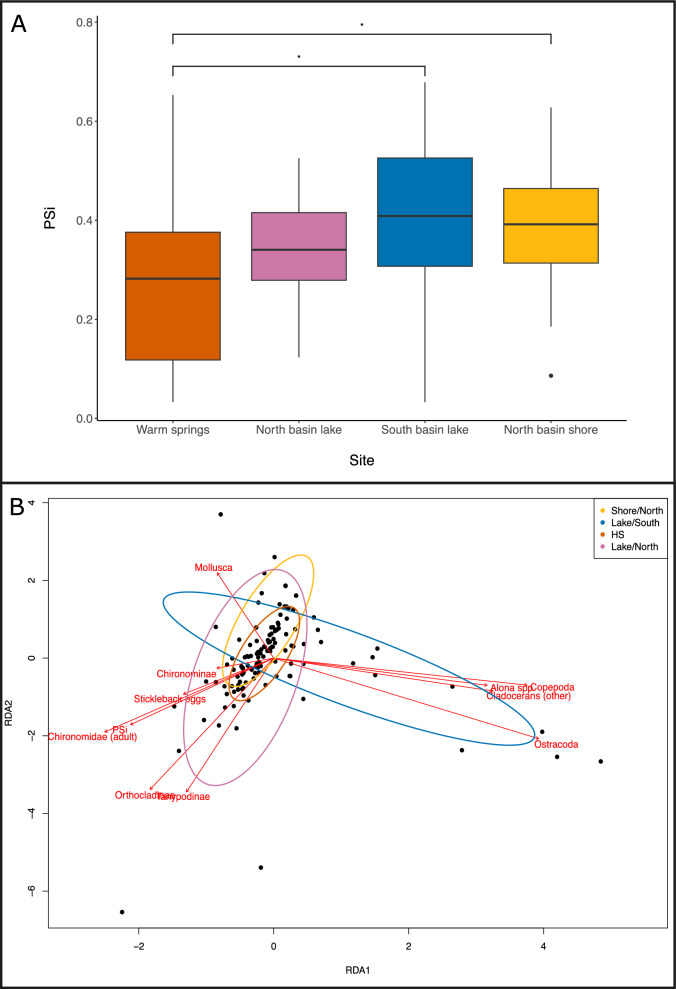
**a** Boxplot showing differences in individual specialisation (Psi) between sites across the lake with significant differences identified in the model indicated by horizontal lines and asterix, and **b** plot showing the first two axes from an ordination analysis used to explore the covariance between diet and Psi where points are individuals and coloured ellipses cluster individuals based on the location at which they were caught

We found that whilst some individuals specialized in their diet relative to the population’s dietary niche, the overall extent of specialization across the lake was quite low, as indicated by the lake-wide and regional WIC/TNW ratios generally exceeding 0.70 (Table 3). This suggests that most individuals fed on a variety of food items present within the population’s dietary niche, without specializing on certain food types. The warm spring site was an exception, with a much lower WIC/TNW ratio and average *PS*_*i*_ than the other sites (Fig. 1B and Fig. 6), suggesting greater individual specialization. The RDA revealed clear associations between individual specialization and particular diet items (Fig. 1B and Fig. 6). Specifically, generalists (i.e., individuals with a higher *PS*_*i*_) tended to feed on chironomids and stickleback eggs, while specialists tended to feed on either crustaceans (e.g., copepods or cladoceran’s) or mollusks. This is consistent with chironomids being the most widely represented diet item, with 54% of individuals consuming chironomids of at least one type (Fig. 1B). Moreover, mollusks were the most prevalent diet item among fish from the warm springs, but were found only in 25% of individuals (Fig. 1B), reflecting the relatively high degree of specialization at that site. There was very little evidence to suggest that *PS*_*i*_ was associated with any morphological trait (Table 4). However, although there was only limited statistical evidence, PC3 had the strongest (negative) association with *PS*_*i*_ among the traits we considered (Table 4), suggesting that individuals with a relatively smaller and more open operculum were more likely to have a negative *PS*_*i,*_ and therefore had a more specialized diet. We found evidence to suggest that males were less specialized than females, indicated by males having a higher value for *Psi* than females (Table 4).

**Table 3:** Total dietary niche width (TNW) across the whole lake and within each site together with the within individual (WIC) and between individual components (BIC) for each site. Ratio of WIC/TNW indicates the extent of individual dietary specialisation in that set of individuals

	Full lake	Hot shore	Shore north	Lake north	Lake south
TNW	767.35	77.71	209.46	2869.66	121.89
BIC	204.95	63.57	54.94	562.17	46.08
WIC	562.40	14.14	154.51	2307.49	75.82
WIC/TNW	0.73	0.18	0.74	0.8	0.62
Number of individuals	144	27	55	24	38
Mean number of diet items in stomach (standard deviation)	32.53 (54.34)	9.78 (14.22)	29.53 (27.66)	82.92 (108.16)	21.21 (26.69)

**Table 4:** Summary table of GLMM used to test how morphology and site predict PSi (as a measure of individual specialisation). Parameter estimates correspond to posterior medians, with 95% posterior uncertainty intervals (based on quantiles) in parenthesis. Continuous predictor variables were z-scored before model fitting, such that estimates represent effect sizes on that scale

	Parameter	Estimate
Fixed effects	Intercept	0.334 (0.211; 0.452)
	Body length	0.015 (− 0.012; 0.042)
	Gut length	0.015 (− 0.012; 0.041)
	Gill raker length	0.009 (− 0.014; 0.032)
	Gill raker gap width	0 (− 0.022; 0.021)
	PC1	0.254 (− 0.193; 0.696)
	PC2	− 0.049 (− 0.558; 0.471)
	PC3	− 0.826 (− 1.834; 0.171)
	Sex_M_	0.052 (− 0.003; 0.107)
Random effects (SD)	Site	0.134 (0.035; 0.36)
	Residual	0.147 (0.132; 0.165)

## Discussion

In this study, we sampled stickleback across Lake Mývatn to examine spatial variation in diet, its relationship with morphology, and the degree of individual specialization in dietary niche. Consistent with previous findings (Millet et al. 2013; Strickland et al. 2023), we found that functional traits such as body length, gut length, and head shape varied across the lake. Diet composition also varied spatially and was associated with key morphological traits, particularly body size and gut length. While individuals in the population were largely generalist feeders, individual specialization was more pronounced in the shoreline fed by warm springs. These results indicate that the dietary niches of Mývatn stickleback vary along environmental gradients and morphological axes, which might contribute to ecological interactions and evolutionary trajectories in this population.

Individual variation in diet was structured in part by environmental heterogeneity across the lake. While copepods and chironomids were relatively ubiquitous in stickleback diets across most sites, other taxa showed stronger spatial differentiation. Mollusks, for example, were most heavily consumed at the warm springs site, a shallow embayment off the lake’s northeastern edge. Stickleback eggs were most prevalent in diets at shoreline sites, particularly among males. Other prey types showed more variable distributions that did not clearly follow basin or shore proximity. These patterns may arise from both selective feeding and spatial differences in prey availability. Mollusks, for instance, are thought to prefer the shallow, silty habitats characteristic of the warm springs area (Bespalaya et al. 2021). Similarly, the high prevalence of stickleback eggs in diets of shoreline stickleback, especially in males, may relate to behavioral and reproductive strategies whereby male stickleback often nest in shallow habitats and can engage in egg cannibalism as part of courtship (Rohwer 1978). Indeed, in other populations of stickleback, nesting in shallower habitats has been linked to greater male breeding success, and nesting depth choice is thought to be under strong sexual selection, potentially involving increased egg cannibalism (Bolnick et al. 2015). Thus, spatial diet variation may reflect not only prey distributions but also microhabitat choice. Given that the Mývatn stickleback population is highly panmictic with extensive dispersal between basins (Strickland et al. 2023; Phillips et al. 2023), we suggest that the spatial dietary variation likely arises from within-generation differences in habitat and niche selection rather than long-term local adaptation in combination with differences in food availability across the different habitats.

Dietary differences among individuals were also associated with some key phenotypic traits, especially body size and gut length. Smaller individuals tended to feed on small food items (e.g., Cladocera), while larger individuals consumed a broader range of prey, including some of the largest prey items such as mollusks and Chironomids. This supports the well-established link between body size and trophic interactions across taxa (Cohen et al. 1993; Brose et al. 2006; Gravel et al. 2013). Moreover, while our analysis was putatively restricted to adults, dietary differences across body size may also reflect ontogenetic niche shifts (Werner and Gilliam 1984; Sillett and Foster 2000; Sánchez-Hernández et al. 2022).

Individuals with relatively longer guts fed on more hard-bodied prey such as mollusks and crustaceans (except for *Alona spp.*) but fewer stickleback eggs. This pattern is intuitive as longer guts are associated with greater capacity for digesting tougher food items (Duque-Correa et al. 2024). However, the direction of causality is not entirely clear. Gut length may constrain diet choice, or conversely, diet may influence gut development over time. More specifically, gut length may constrain individuals to feed on food items of a particular digestibility, or alternatively the diet that individuals consume may shape the development of the gut (Olsson et al. 2007; Wagner et al. 2009; Davis et al. 2013). It is likely that the relationship between gut length and diet found here is the result of a combination of both constraints caused by genetic component of the trait and diet-induced plasticity of the trait. Further work would needed to specifically disentangle these effects, for instance with common garden and plasticity experiments (Lundsgaard-Hansen et al. 2013). Studying diet-induced changes in phenotypes could shed light on how ecology, development, and natural selection might intersect to shape the length of the gut (Skúlason et al. 2019).

The observed patterns of trait-diet associations could also reflect either the matching habitat choice hypothesis whereby individuals feed on diet that corresponds to their morphology, or adaptive plasticity in response to a temporally fluctuating environment, rather than fixed genetic adaptation. Morphological variation driven by diet is known to depend on the specific prey or habitat types available (Green and Côté 2014), and plastic responses allow individuals to rapidly adjust to environmental shifts, such as changes in prey abundance, within or across generations (Pfennig 1990; Crispo 2008; Vervust et al. 2010; Van Kleeck et al. 2015). For instance, a temporary decline in chironomid populations in lake Mývatn, as noted by (Ives et al. 2008), could prompt stickleback to shift their diet toward cladocerans, resulting in morphological changes that improve feeding efficiency on the alternative prey (Day and McPhail 1996). As noted above, these responses may be plastic or reflect a genetic response to fluctuating selection but in a high gene flow population like this, plasticity in particular may serve to optimize fitness across varying conditions rather than promoting disruptive selection for fixed specialist morphs, thus facilitating rapid but reversible adaptation to environmental variability. Critically, whichever of these mechanisms are at play, the resulting morphological composition of the stickleback population may cause feedback on the prey composition of the lake, causing eco-evolutionary feedback loops between stickleback morphology and prey composition. One would need to run multi-generation mesocosm experiments to explicitly disentangle such relationships (Skúlason et al. 2019).

Despite the overall pattern of generalist feeding, we observed some evidence for individual specialization, particularly among individuals consuming either mollusks or crustaceans. However, we did not detect strong associations between specific morphological traits and individual specialization, which is perhaps unsurprising given the different ways in which individuals can specialize (i.e., individuals can specialize on different types of food). Nonetheless, the overall tendency towards generalized diets in this population is striking, especially given previous reports of strong individual specialization in other stickleback populations (Bolnick and Paull 2009; Matthews et al. 2010; Bolnick and Ballare 2020). Most notably, prior studies have often described divergence along a benthic–limnetic axis, with specialization on limnetic prey associated with longer, more closely spaced gill rakers (McGee et al. 2013). Our results suggest a slightly different pattern, characterized by a chironomid–crustacean axis, with mollusks contributing a third dietary dimension in the warm springs, that echoes but does not exactly match the previously established pattern. Mývatn’s shallow depth and high benthic productivity may limit the potential for benthic-limnetic differentiation and instead promote more complex axes of trophic variation.

Interestingly, we observed greater individual specialization in the warm springs site, characterized by warmer water than in the rest of the lake (Millet et al. 2013; Strickland et al. 2023). This pattern may emerge as a result of a number of factors which differentiate this area over the rest of the lake, including a more stable thermal environment, different prey community composition and high stickleback density (Einarsson et al. 2004; Phillips et al. 2023; Strickland et al. 2023). These features may indicate that the increased metabolic demands of living in warm water coupled with strong local competition emerging as a result of high density promotes niche partitioning, even within a largely generalist population. Higher stickleback density likely plays a particularly important role as prior work shows that high intraspecific competition leads to individual specialization, because specializing on specific parts of the populations dietary niche can function to reduce intraspecific competition (Svanbäck and Bolnick 2006; Snowberg et al. 2015). However, specialization may involve trade-offs that reduce flexibility in exploiting other prey types (Svanbäck and Bolnick 2006), which might be disadvantageous in a system like Mývatn where both stickleback and prey populations fluctuate over time (Einarsson et al. 2004; Ives et al. 2008; Phillips et al. 2023). A greater degree of specialization at the warm springs site could therefore suggest that, together with the higher density of stickleback, prey availability may be more predictable at this site, but more work to measure the prey community composition through time would be needed to test this hypothesis.

There is a substantial body of research on dietary niche variation and individual specialization in threespine stickleback, especially in North American populations (Bolnick and Paull 2009; Matthews et al. 2010; Bolnick and Ballare 2020). As a model species in the study of parallel evolution (Hendry et al. 2013; Reid et al. 2021), stickleback provide insights into the mechanisms driving ecological divergence. Our study adds valuable data from an underrepresented geographic region and highlights how fine-scale spatial and ecological variation can shape trophic strategies. Notably, our use of high-resolution dietary data, without aggregating prey taxa into broad functional groups, allowed us to detect nuanced differences in prey use that might underscore the low levels of specialization observed here. However, our findings do reflect a single time point, and both diet and specialization may vary seasonally or across years. Future studies that incorporate temporal sampling would provide a clearer picture of how stable these patterns are over time. Nevertheless, our results underscore the importance of ecological heterogeneity in generating and maintaining diversity in diet and morphology within a single, highly connected population.

## Supplementary Information

Below is the link to the electronic supplementary material.

Supplementary file1 (DOCX 955 KB)

## Data Availability

All data and code needed to reproduce results presented in this manuscript can be found at 10.5281/zenodo.19553725.
