# Supplementary material for: Spatial variation and individual specialization of stickleback diet in relation to trophic morphology
Source: Aquat Ecol. 2026 Apr 24;60(2):55. doi: 10.1007/s10452-026-10299-x (PMC13109291; doi:10.1007/s10452-026-10299-x)
Supplement: Supplementary file 1 — Supplementary file1 (DOCX 955 KB) [file 10452_2026_10299_MOESM1_ESM.docx]

**Spatial variation and individual specialization of stickleback diet
in relation to trophic morphology**

Snorradóttir et al.

Supplementary materials

**Figure S1.** First two axes of RDA ordination describing covariance in diet items fed on by individual stickleback with ellipses grouping individuals from the same site.

**Table S1.** Prevalence (proportion of fish where taxon was present) and mean abundance for each taxon across all samples. Some taxa were grouped together for analysis to ensure reasonable representation across samples.

| Taxon (finest resolution) | Revised taxon for analysis (if modified) | Prevalence | Mean abundance (standard deviation) |
| --- | --- | --- | --- |
| Adult chironomid |  | 0.44 | 2.74 (5.75) |
| Orthocladiinae |  | 0.29 | 2.40 (9.38) |
| Chironomini | Chironominae | 0.28 | 1.18 (2.87) |
| Unknown *Alona spp.* | *Alona spp.* | 0.22 | 3.81 (20.2) |
| Adult copepod | Copepod | 0.21 | 6.47 (29.4) |
| Tanypodinae |  | 0.12 | 0.41 (1.87) |
| *Alona affinis* or  *Alona quadrangularis* | *Alona spp.* | 0.11 | 0.89 (4.47) |
| Stickleback eggs |  | 0.10 | 0.86 (4.02) |
| *Alona rectangula* | *Alona spp.* | 0.08 | 0.41 (2.43) |
| Gastropod | Mollusk | 0.07 | 0.16 (0.74) |
| Ostracod |  | 0.05 | 0.31 (1.71) |
| *Alonella* *nana* | Cladoceran | 0.05 | 0.59 (5.00) |
| Bivalve | Mollusk | 0.03 | 0.05 (0.35) |
| Juvenile copepod | Copepod | 0.03 | 0.05 (0.31) |
| Unknown chironomid larvae | (Omitted) | 0.02 | 0.03 (0.20) |
| *Acroperus harpae* | Cladoceran | 0.02 | 0.05 (0.37) |
| *Macrothrix hirsuticornix* | Cladoceran | 0.01 | 0.02 (0.17) |
| *Daphnia longispina* | Cladoceran | 0.01 | 0.07 (0.85) |
| Cladoceran resting egg | (Omitted) | 0.01 | 0.02 (0.29) |
| Tanytarsini | Chironominae | 0.01 | 0.01 (0.10) |

**Figure S2.** Scatter plot of number of the observed diet items for each taxon as a function of body length.

**Figure S3.** Scatter plot of number of the observed diet items for each taxon as a function of gill raker length.

**Figure S4.** Scatter plot of number of the observed diet items for each taxon as a function of gap width.

**Figure S5.** Scatter plot of number of the observed diet items for each taxon as a function of gut length.

**Figure S6.** Scatter plot of number of the observed diet items for each taxon as a function of PC1.

**Figure S7.** Scatter plot of number of the observed diet items for each taxon as a function of PC2.

**Figure S8.** Scatter plot of number of the observed diet items for each taxon as a function of PC3.

**Figure S9.** Variation in the zero-inflated probability for each taxon estimated with a multivariate GLMM. Points indicate posterior medians of the estimated relative trait value with the error bars depicting 68% and 95% posterior uncertainty intervals (based on quantiles; 68% intervals match the coverage of standard errors).
